# Activation of the NLRP3 Inflammasome Complex is Not Required for Stress-Induced Death of Pancreatic Islets

**DOI:** 10.1371/journal.pone.0113128

**Published:** 2014-11-18

**Authors:** Jibran A. Wali, Esteban N. Gurzov, Stacey Fynch, Lorraine Elkerbout, Thomas W. Kay, Seth L. Masters, Helen E. Thomas

**Affiliations:** 1 St Vincent’s Institute, Fitzroy, Victoria, Australia; 2 The University of Melbourne Department of Medicine, St. Vincent’s Hospital, Fitzroy, Victoria, Australia; 3 The Walter and Eliza Hall Institute of Medical Research, Parkville, Victoria, Australia; University of California Merced, United States of America

## Abstract

Loss of pancreatic beta cells is a feature of type-2 diabetes. High glucose concentrations induce endoplasmic reticulum (ER) and oxidative stress-mediated apoptosis of islet cells *in* *vitro*. ER stress, oxidative stress and high glucose concentrations may also activate the NLRP3 inflammasome leading to interleukin (IL)-1β production and caspase-1 dependent pyroptosis. However, whether IL-1β or intrinsic NLRP3 inflammasome activation contributes to beta cell death is controversial. This possibility was examined in mouse islets. Exposure of islets lacking functional NLRP3 or caspase-1 to H_2_O_2,_ rotenone or thapsigargin induced similar cell death as in wild-type islets. This suggests that oxidative or ER stress do not cause inflammasome-mediated cell death. Similarly, deficiency of NLRP3 inflammasome components did not provide any protection from glucose, ribose or gluco-lipotoxicity. Finally, genetic activation of NLRP3 specifically in beta cells did not increase IL-1β production or cell death, even in response to glucolipotoxicity. Overall, our results show that glucose-, ER stress- or oxidative stress-induced cell death in islet cells is not dependent on intrinsic activation of the NLRP3 inflammasome.

## Introduction

There is evidence from human studies and animal models that loss of pancreatic beta cell mass occurs in type 2 diabetes [1]–[5]. Histological examination of pancreatic specimens from type 2 diabetic individuals showed a reduction in beta cell mass and an increase in the number of terminal deoxynucleotidyl transferase dUTP nick end labelling (TUNEL) positive beta cells compared to non-diabetic individuals [1]–[3]. Elevated plasma glucose is a hallmark of diabetes, and chronic exposure to high concentrations of glucose *in* *vitro* causes apoptosis (glucose toxicity) of islet cells [6]–[9]. We have demonstrated that apoptosis induced by glucose is due to activation of the intrinsic apoptosis pathway [6]. The pro-apoptotic BH3-only proteins BIM and PUMA, and downstream effector molecule BAX are important mediators of glucose toxicity [6]. Expression of pro-apoptotic molecules including BIM, PUMA and BAX was observed in islets isolated from subjects with type 2 diabetes [10], [11].

It has been reported that exposure of mouse or human islets to high glucose concentrations induces production of IL-1β that could be toxic for islet cells [9], [12]–[14]. IL-1β is produced as a result of activation of the NLRP3 inflammasome. This protein complex comprises of NLRP3, the adaptor protein ASC and caspase-1 [15]. Activation of the NLRP3-inflammasome requires two signals. Signal-1 increases intracellular pro-IL-1β concentration in response to binding of ligands to toll-like receptors (TLR). For most *in* *vitro* studies, lipopolysaccharide (LPS) is used to provide signal-1 whereas substances such as minimally modified LDL and free fatty acids could possibly serve this role *in* *vivo*. Signal-2 causes activation of the NLRP3-inflammasome resulting in generation of active caspase-1. Caspase-1 cleaves pro-IL-1β to IL-1β that can then be secreted by the cell. Substances such as cholesterol crystals, nigericin, alum and uric acid crystals have been shown to activate the inflammasome [14], [16]–[18].

Some substances can provide both signal-1 and 2. These include glucose, ER stress-inducing drugs such as thapsigargin and tunicamycin, and the mitochondrial oxidative stress-inducing drug rotenone [13], [19], [20]. It has been suggested that increased reactive oxygen species (ROS) accumulation inside the cell causes dissociation of the thioredoxin-interacting protein (TXNIP) from the antioxidant protein thioredoxin resulting in its activation. TXNIP then binds to NLRP3 to stimulate its activation [13], [20]. In addition to IL-1β mediated apoptosis, inflammasome activation can lead to caspase-1-dependent death of the IL-1β-producing cell called pyroptosis. Pyroptosis is characterised by DNA fragmentation, cellular swelling and formation of pores in the plasma membrane [15].

Activation of ER stress molecules IRE1α and PERK, as well as high concentrations of glucose can also activate the inflammasome via TXNIP upregulation [9], [12], [13], [19], [21]. However, the involvement of TXNIP in inflammasome activation is controversial. While suppression of TXNIP in the INS-1 beta cell line reduced thapsigargin toxicity [21], mouse islets lacking TXNIP were not protected across a range of concentrations tested [22].

Macrophages express inflammasome components including NLRP3, ASC and caspase-1, and show inflammasome activation, in response to pathogens [18], [23], [24]. However, while their expression in pancreatic islets has been demonstrated, it is at much lower levels when compared with bone marrow derived macrophages [13], [25]. In addition, although the expression of TLR4 and IL-1β has been reported previously in human and mouse islets and MIN6 beta cells [25]–[29], inflammasome activation has not convincingly been demonstrated in beta cells. In addition, a recent study reported absence of TLR4 in rat beta cells, making the understanding of the role for these factors in beta cells more complicated [29]. Therefore, we decided to directly examine whether deletion of inflammasome components in islets results in protection from cell death. Our results show that deletion of inflammasome components does not affect the apoptosis of islet cells in response to glucose or chemicals that induce oxidative or ER stress, and that activation of NLRP3 in beta cells does not contribute to IL-1β production and islet cell death.

## Methods

### Mice

Mice deficient in caspase-1 were obtained from Jackson Laboratories on a NOD/Lt background (NOD.129S2(B6)-*Casp1^tm1Sesh^*/LtJ) and backcrossed onto the C57BL/6 genetic background for 10 generations by Dr Ben Croker (WEHI). NLRP3 hypomorphic mutant mice (Nlrp3^A350Vne^°^R^) were generated by Dr Susannah Brydges and Dr Hal Hoffman (UCSD) [30]. These mice are effectively a loss of function mutation when bred as homozygous mutants without Cre recombinase. For simplicity of nomenclature, these mice have been called NLRP3^−/−^ in this article. NLRP3^A350V/A350V^ mutant mice were bred with RIP-Cre from Jackson Laboratories (B6.Cg-Tg(Ins2-cre)^25Mgn^/J) to generate mice with gain of function NLRP3 activity in the pancreatic beta cells. All animal experiments were carried out in compliance with the ‘Australian code for the care and use of animals for scientific purposes’ and were approved by the St Vincent’s Hospital Animal Ethics Committee (AEC protocol number 016/11).

### Reagents

D-glucose (used at 33.3 mM) and D-ribose (50 mM) were purchased from Invitrogen (Invitrogen Corporation, Grand Island, New York) and Sigma-Aldrich (St Louis, MO) respectively. Similar concentrations of L-glucose or L-ribose were used as hyperosmolarity controls (data not shown) [6]. Thapsigargin (used at 5 µM) was purchased from Calbiochem (Darmstadt, Germany) and IL-1Ra (used at 5 µg/mL) was obtained from Amgen (Thousand Oaks, CA) [31]. Hydrogen peroxide (H_2_O_2_, Sigma-Aldrich) was used at 0–40 µM, Rotenone (Sigma-Aldrich) at 100 nM and lipopolysaccharide (LPS, Sigma-Aldrich L2630, from *Escherichia coli* 0111:B4) at 100 nM. These reagents were tested across a range of concentrations in wild-type islets to determine the optimal concentration to use for further experiments. Control conditions contained the same concentration of diluent (DMSO for rotenone and ethanol for thapsigargin) in complete medium. Palmitate (Sigma-Aldrich) was used at 1 mM coupled to fatty acid free 1% BSA (Roche, Mannheim, Germany) and was added to 10% serum containing medium for islet treatment.

### Islet isolation, culture and DNA fragmentation assay

Islets of Langerhans were isolated as described previously [32]. Islets were washed, hand-picked, and cultured overnight at 37°C in 5% CO_2_ in CMRL medium-1066 (Invitrogen) supplemented with 100 units/mL penicillin, 100 µg/mL streptomycin, 2 mmol/L glutamine, and 10% FCS (JRH Biosciences, Lenexa, KS) (referred to below as complete CMRL). CMRL medium contains 5.5 mM glucose that is similar to the glucose concentrations islets are exposed to *in* *vivo*. Following incubation with different reagents, 100 islets per sample were trypsinized and DNA fragmentation was analyzed by staining with propidium iodide and flow cytometric analysis as previously described [6]. NIT-1 cells are derived from transgenic NOD mice expressing the SV40 large T antigen in pancreatic beta cells under control of the rat insulin promoter [33]. Cells were maintained in 10% CO_2_ at 37°C in DMEM (Invitrogen) supplemented with non-essential amino acids, antibiotics and 10% FCS.

### PCR genotyping

DNA was prepared from purified islets, and subjected to PCR with the following primers: Nlrp3^A350V^F: CCCTGCATTTTGTTGTTGTTG, Nlrp3^A350V^R: CCTGCTTCTCACATGTCGTC, NeoF: GAAGCGGGAAGGGACTGGCTGCTA and NeoR: CGGGAGCGGCGATACCGTAAAGC.

### ELISA

After isolation, islets were cultured overnight at 37°C in complete CMRL medium. Next day, 400 islets/sample were hand-picked and cultured in 1 mL of complete CMRL medium for 2.5 days. Alternatively, macrophages were prepared by culturing mouse bone marrow in M-CSF for 7 days, re-plated at 10^5^ per well in 96 well plates, followed by stimulation with palmitate conjugated to BSA overnight. At the conclusion of culture, the supernatant was removed and stored at −80°C for later use. ELISA for IL-1β was performed according to manufacturers instructions (R & D Systems, Minneapolis, MN).

### Western blotting

Four hundred islets/sample were cultured in 1 mL of 1% BSA containing complete CMRL medium and treated with a combination of LPS (100 nM), glucose (33.3 mM) and palmitate (1 mM) for 2.5 days. Control islets were cultured in the same medium containing 100 nM LPS. Islet lysates were then prepared in RIPA buffer as described previously [34]. Macrophages were also treated with the same concentration of LPS, glucose and palmitate for 24 h then lysed directly in reducing sample buffer. Western blotting was performed using anti-caspase-1 (p20) from Adipogen (Casper-1, Adipogen International, San Diego, CA).

### Lactate dehydrogenase (LDH) assay

Islets were cultured and treated as described above for the western blotting experiments. Supernatants were obtained at the end of culture and LDH concentrations were determined. A commercially available kit from Roche was used for LDH assay.

### Statistical analysis

Statistical analysis was performed using GraphPad Prism Software (San Diego, CA). All data shown as bar graphs are mean + SEM. Data were analysed by one-way or two-way ANOVA with Bonferroni’s or Dunnett’s post test for comparison of multiple columns (as appropriate).

## Results

### Oxidative and ER stress do not cause NLRP3 inflammasome mediated islet cell death

#### Oxidative stress induced cell death is not inflammasome mediated

**Figure 1:**
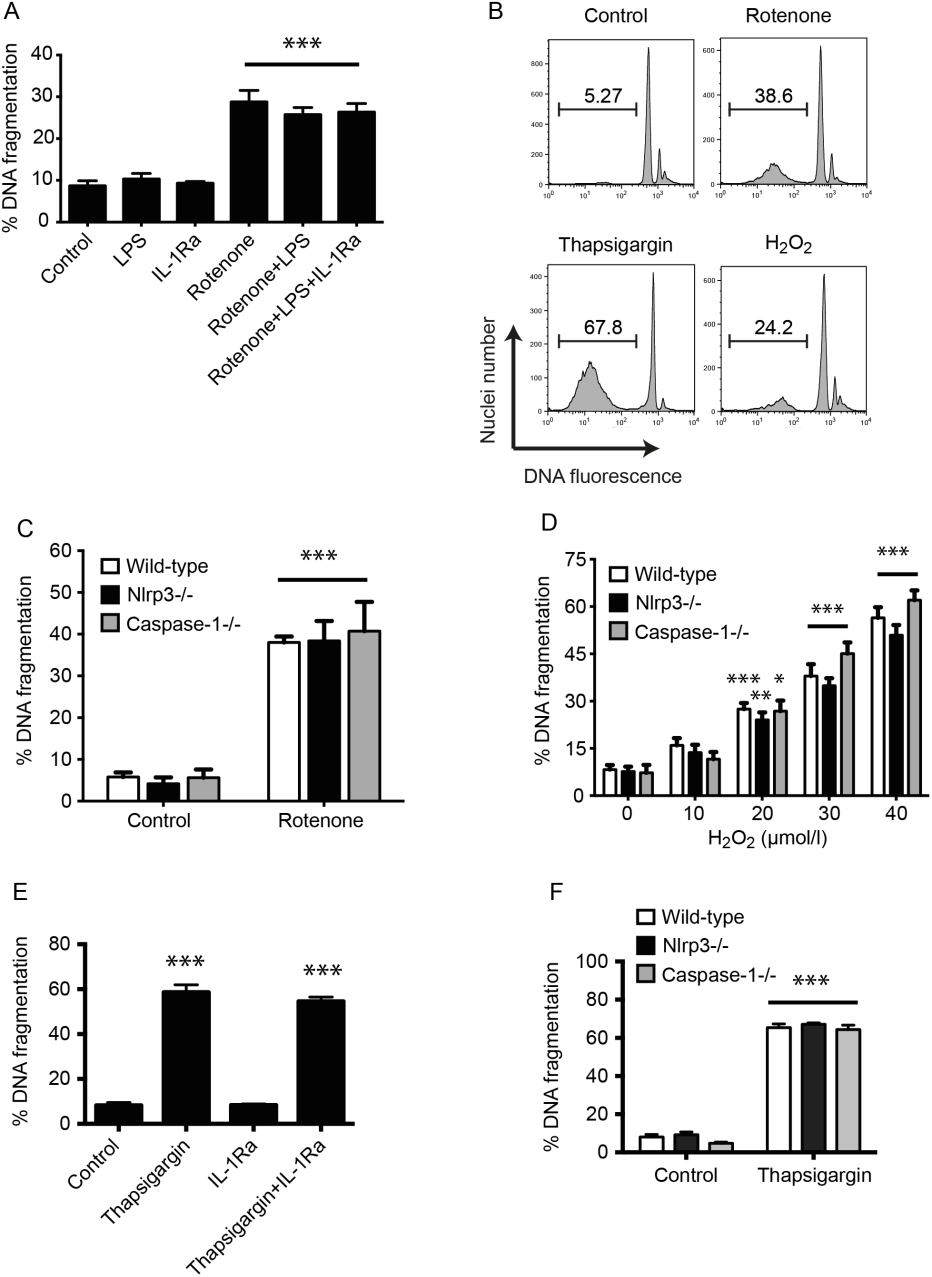
Oxidative and ER stress induced islet cell death is not mediated by inflammasome activation. (**A**) NIT cells were cultured in control medium, or medium containing 100 ng/mL LPS, 5 µg/mL IL-1Ra or 100 nM rotenone for 2 days. The frequency of cells undergoing DNA fragmentation was measured by flow cytometry. Results are mean+SEM of n≥3 independent experiments. ***p<0.001 Rotenone treated NIT cell groups vs controls without rotenone. (**B**) Representative FACS profiles of DNA fragmentation in islets after treatment with 100 nM rotenone for 2 days, 20 µM H_2_O_2_ for 2 days or 5 µM thapsigargin for 5 days. The percentage of islet cells with fragmented nuclei is indicated. (C) C57BL/6, Nlrp3^−/−^ or caspase-1^−/−^ islets were cultured in control medium or medium containing 100 nM rotenone for 2 days. Control islets were incubated in medium containing an equal volume of solvents. Results are mean+SEM of n≥3 independent experiments. ***p<0.001 rotenone treated islets vs controls of same genotype. (**D**) DNA fragmentation was measured by flow cytometry after incubation of wild-type C57BL/6, Nlrp3^−/−^ or caspase-1^−/−^ islets with 0–40 µM H_2_O_2_ for 2 days. Results are mean+SEM of n≥3 independent experiments. *p<0.05, **p<0.01, ***p<0.001 H_2_O_2_ treated islets vs untreated islets of same genotype. (**E, F**) Wild-type islets (E), or wild-type, Nlrp3^−/−^ or caspase-1^−/−^ islets (F) were cultured in control medium, or medium containing 5 µg/mL IL-1Ra or 5 µM thapsigargin for 5 days. Control islets were incubated in medium containing an equal volume of solvents. The frequency of cells undergoing DNA fragmentation was measured by flow cytometry. Results are mean+SEM of n≥3 independent experiments. ***p<0.001 Thapsigargin treated islets vs controls without thapsigargin of same genotype. No significant differences were observed between the different genotypes.

Oxidative stress has been shown to cause activation of the NLRP3 inflammasome [20]. In particular, there is evidence that mitochondrial ROS is a potent activator of the NLRP3 complex in non-islet cells [20]. We studied the role of inflammasome activation in mitochondrial ROS mediated cell death in both beta cell lines and primary islets. Oxidative stress-sensitive NIT-1 beta cells were treated with 100 nM rotenone to induce mitochondrial ROS production. Although rotenone is able to provide both signal 1 and 2, we nevertheless pre-treated cells with 100 ng/mL LPS to maximally increase cellular stores of pro-IL-1β. Cell death was quantified by DNA fragmentation, which is common to all known forms of programmed cell death [15]. Inhibition of IL-1β activity with the receptor antagonist for IL-1β (IL-1Ra, 5 µg/mL) did not provide any protection from rotenone induced DNA fragmentation. This shows that mitochondrial ROS does not cause IL-1R-dependent death of beta cells (Figure 1A).

It remains possible that inflammasome activation could occur in islet resident macrophages or other non-beta cells, resulting in IL-1β secretion and toxicity to beta cells. Therefore, islets isolated from wild-type, Nlrp3^−/−^ and caspase 1^−/−^ mice were exposed to rotenone for 2 days. DNA fragmentation was similar in wild-type and NLRP3 or caspase-1 deficient islets suggesting that mitochondrial oxidative stress causes islet cell death through inflammmasome independent pathways (Figure 1B & C).

To further investigate the possible contribution of oxidative stress, islets from both Nlrp3^−/−^ and Caspase-1^−/−^ mice were exposed to cytosolic ROS by culturing them with hydrogen peroxide. Hydrogen peroxide-induced DNA fragmentation was similar in wild-type and knock-out islets, indicating that the NLRP3 inflammasome is not a mediator of islet cell death induced by cytosolic ROS (Figure 1D).

#### ER stress-induced death is not inflammasome mediated

To examine if ER stress could cause inflammasome mediated islet cell death, wild-type islets were treated with thapsigargin and IL-1Ra. The addition of IL-1Ra did not protect islets from thapsigargin toxicity (Figure 1B & E) indicating that ER stress induced islet cell death is not mediated by IL-1β secretion. For further confirmation, Nlrp3^−/−^ and caspase-1^−/−^ islets were incubated with 5 µm thapsigargin for 5 days. No difference in DNA fragmentation was observed between wild-type and Nlrp3^−/−^ or caspase-1^−/−^ islets, demonstrating that ER stress-induced islet toxicity does not involve activation of the NLRP3 inflammasome complex (Figure 1F).

### A high concentration of glucose does not cause NLRP3 inflammasome mediated islet cell death

**Figure 2:**
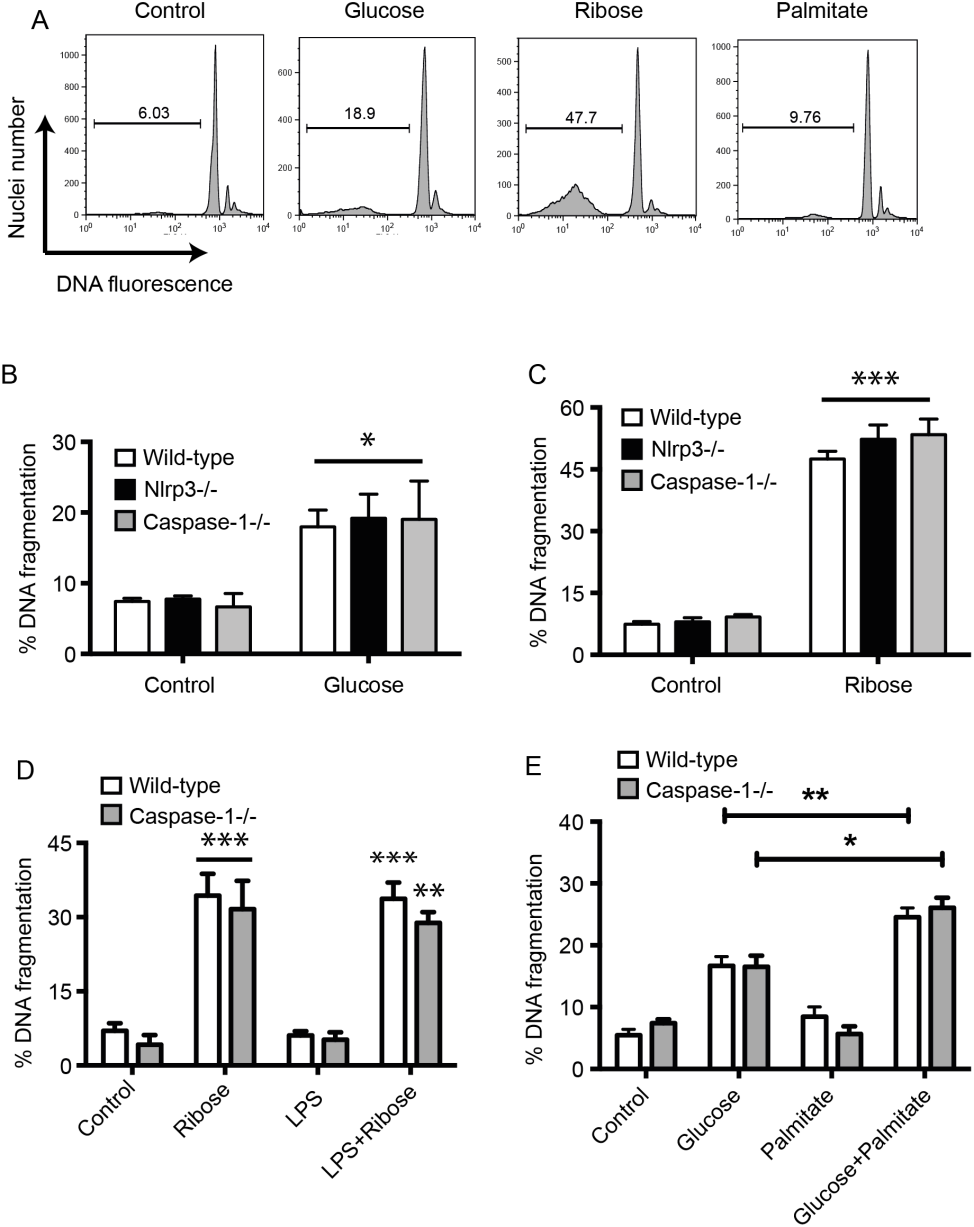
Glucose toxicity of islet cells is not mediated by inflammasome activation. (A) Representative FACS profiles of DNA fragmentation in islets after treatment with 33.3 mM glucose for 6 days, 50 mM ribose for 4 days or 1 mM palmitate conjugated to 1% BSA for 6 days. The percentage of islet cells with fragmented nuclei is indicated. (B, C) DNA fragmentation was measured by flow cytometry after incubation of wild-type C57BL/6, Nlrp3**^−/−^** or caspase-1**^−/−^** islets with 33.3 mM glucose for 6 days (B) or 50 mM ribose for 4 days (C). Control islets were incubated in medium containing 5.5 mM glucose. Results are mean+SEM of n≥4 independent experiments. *p<0.05 glucose treated islets vs control islets of same genotype. ***p<0.001 ribose treated islets vs control islets of same genotype. (D) DNA fragmentation was measured by flow cytometry after incubation of wild-type or caspase-1**^−/−^** islets with 50 mM ribose or 100 ng/mL LPS for 3 days. Results are mean+SEM of n = 3 independent experiments. **p<0.01, ***p<0.001 ribose treated islets vs controls of the same genotype without ribose. (E) DNA fragmentation was measured by flow cytometry after incubation of wild-type or caspase-1**^−/−^** islets with 33.3 mM glucose or 1 mM palmitate conjugated to 1% BSA for 6 days. Control islets were incubated in a medium containing 5.5 mM glucose and 1% BSA. Results are mean+SEM of n = 3 independent experiments. *p<0.05 glucose vs glucose+palmitate-treated caspase-1**^−/−^** islets, **p<0.01 glucose vs glucose+palmitate-treated wild-type islets.

The possibility that glucose-induced death could be due to inflammasome activation in islets was studied. In addition to glucose, we tested toxicity of islets to the reducing sugar ribose, which is metabolized through the pentose phosphate pathway and glycolysis, and similar to glucose, induces islet cell death through glycation and formation of ROS [35]. Islets lacking functional NLRP3 or caspase-1 were not protected from glucose or ribose toxicity (Figure 2A, B & C). To rule out any possibility of a weak signal-1, islets were also cultured in the presence of LPS to increase the concentration of pro-IL-1β. However, addition of LPS did not increase ribose-induced islet cell death and compared with wild-type islets, we did not see any protection in LPS+ribose treated caspase1^−/−^ islets (Figure 2D).

Whole islets were also treated with 1 mM palmitate (conjugated to 1% BSA in medium containing 10% serum) in the presence or absence of 33.3 mM glucose to study if the combined presence of glucose and palmitate (glucolipotoxicity) is required to cause significant IL-1β secretion. We did not see substantial cell death in whole islets after exposure to palmitate alone, however, exposure of islets to palmitate and glucose together caused greater cell death than each reagent alone. Deletion of caspase-1 from islets did not provide any protection from glucolipotoxicity (Figure 2E). These results, together with our previous findings that deficiency of IL-1 receptors did not protect islets from glucose toxicity [6], show that neither activation of the NLRP3-inflammasome complex, nor IL-1β production mediate islet cell death in response to high concentrations of glucose.

### Beta-cell specific NLRP3 activating mutation does not result in IL-1β production or amplify glucose toxicity

**Figure 3:**
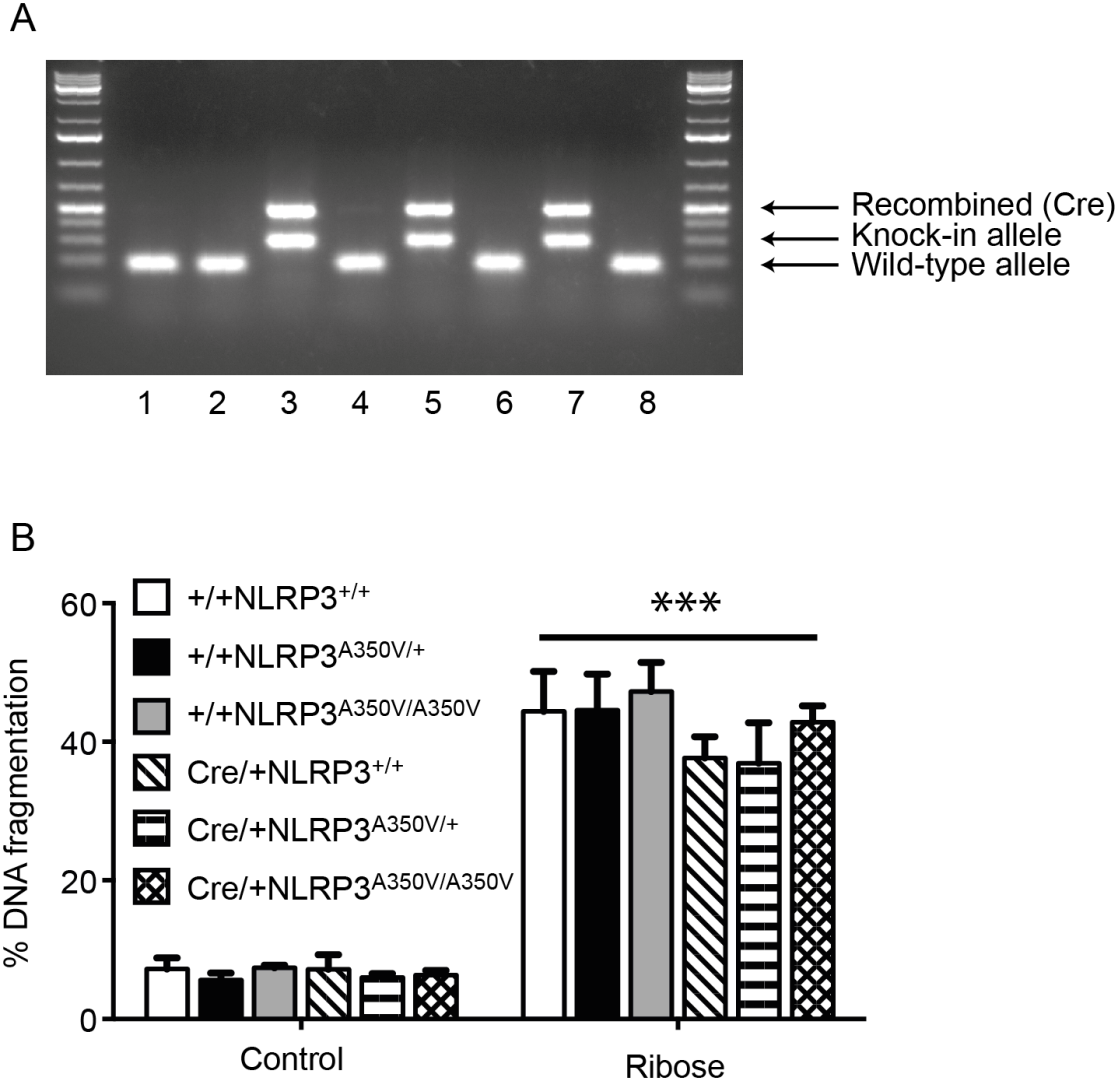
NLRP3 activating mutation does not increase islet glucose toxicity. (**A**) DNA was isolated from 300–500 islets of the indicated genotypes and PCR was performed using appropriate primers. Islets isolated from wild-type (+/+ NLRP3^+/+^) mice were used for lanes 1, 2, 4, 6 and 8, while lanes 3, 5 and 7 show genotyping results for islets expressing mutant NLRP3 (Cre/+ NLRP3^A350V/A350V^). (**B**) Islets from wild-type mice, floxed mice with activating NLRP3 mutation knocked into NLRP3 locus (NLRP3^A350V/+^ or NLRP3^A350V/A350V^), and mice expressing Cre recombinase in beta cells (Cre/+) were treated with 50 mM ribose for 4 days. DNA fragmentation was measured by flow cytometry. Results are mean+SEM of n = 3 independent experiments. ***p<0.001 ribose-treated islets vs control islets of the same genotype.

To determine whether constitutive activation of NLRP3 in beta cells would exacerbate glucose toxicity, we isolated islets from mice with a human NLRP3 activating mutation knocked-in to the mouse NLRP3 locus (NLRP3^A350V/A350V^) [30]. This mutation is controlled by the Cre enzyme, so by crossing the NLRP3^A350V/A350V^ mutant mice to mice expressing Cre under control of the rat insulin promoter (RIP-Cre), we generated mice with mutant NLRP3 expressed at endogenous levels, but only in pancreatic beta cells, denoted NLRP3^A350V/A350V^/RIP-cre. Successful expression of mutant NLRP3 in mouse beta cells was confirmed by PCR using DNA from purified islets (Figure 3A). This mutation is clearly active, as mice crossed to LysM-Cre do not survive beyond 14 days, however NLRP3^A350V/A350V^/RIP-cre mice do not have any overt phenotype (data not shown).

If beta cells are able to produce significant IL-1β in response to glucose or ribose exposure, then islets with constitutive NLRP3 should produce even greater amounts of IL-1β resulting in amplification of glucose or ribose toxicity. Contrary to this expectation, we observed that islets with mutant NLRP3 did not show increased ribose toxicity when compared with controls (Figure 3B).

**Figure 4:**
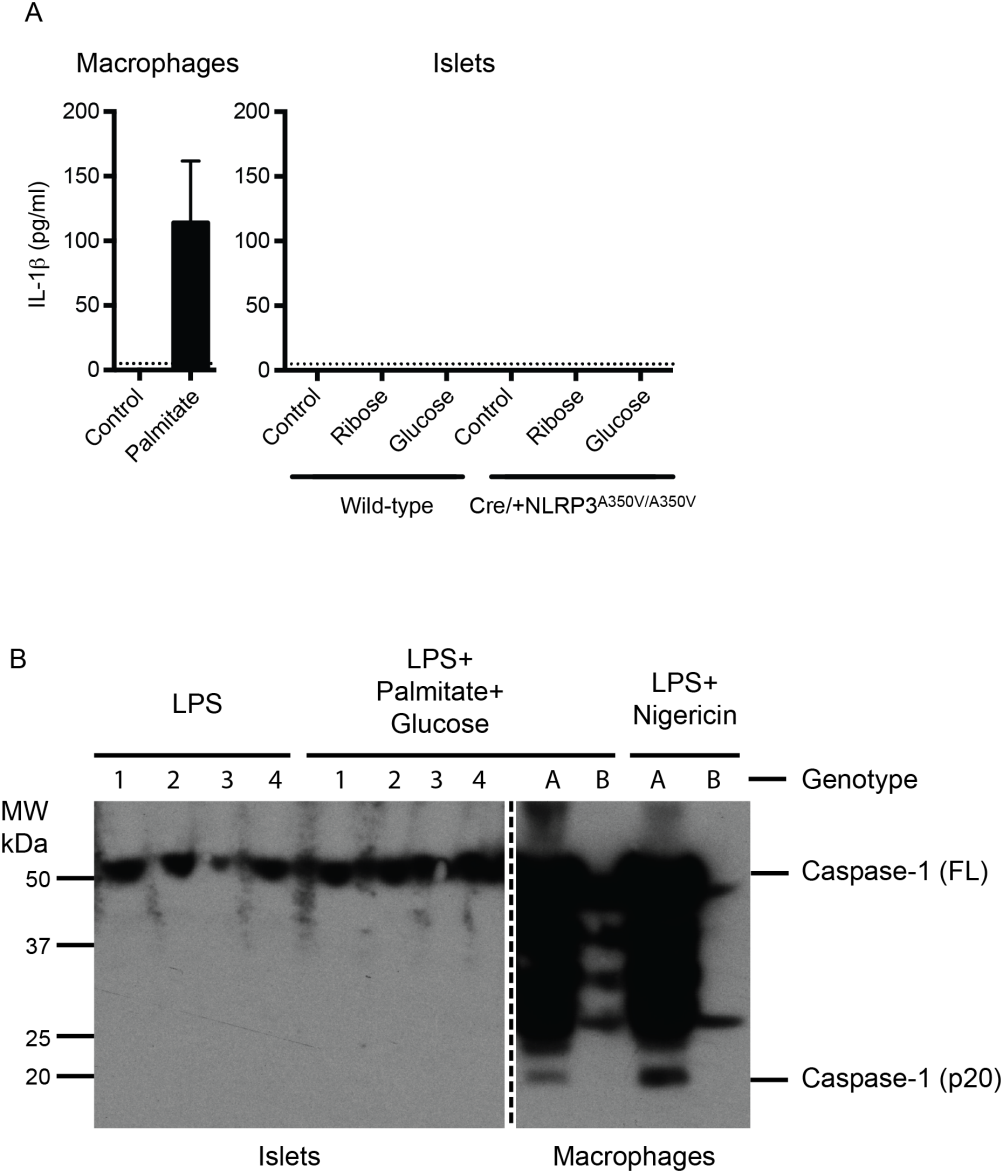
NLRP3 activating mutation does not induce caspase-1 cleavage in islets. (**A**) Four hundred islets isolated from wild-type or Cre/+ NLRP3^A350V/A350V^ mice were cultured in 1 mL of medium containing 33.3 mM glucose or 50 mM ribose for 2.5 days and IL-1β concentration in the supernatant was quantified by ELISA. Control islets were incubated in a medium containing 5.5 mM glucose. Macrophages treated overnight with 1 mM palmitate were used as positive control. Results are mean+SEM of n = 3–4 independent experiments. (**B**) Four hundred islets/sample were cultured in control medium containing 1% BSA and 100 nM LPS or a medium containing 1% BSA, 100 nM LPS, 33.3 mM glucose and 1 mM palmitate for 2.5 days. Lysates were prepared in RIPA buffer and western blotting for caspase-1 (full length, FL or cleaved, p20) was performed. Genotypes 1: +/+ NLRP3^+/+^, 2: Cre/+ NLRP3^+/+^, 3: +/+ NLRP3^A350V/A350V^ and 4: Cre/+ NLRP3^A350V/A350V^. As a positive control, macrophages from wild-type (A) and caspase-1^−/−^ (B) mice were treated with LPS+BSA+Glucose+Palmitate for 24 h or LPS+Nigericin for 1 h.

We measured the IL-1β produced by culturing 400 islets in 1 ml culture medium with 50 mM ribose or 33.3 mM glucose for 2.5 days. It has previously been shown that high concentrations of glucose neither induce IL-1β seretion nor affect its expression in cultured dendritic cells [18]. Therefore, wild-type macrophages cultured with palmitate were included as a positive control. Supernatant was collected and IL-1β concentration was determined by ELISA. Similar to our previous finding [6], we were unable to detect any IL-1β in supernatants from wild-type islets treated with ribose or glucose. In addition, the IL-1β concentration remained below the threshold for detection even in the supernatants from ribose or glucose treated NLRP3 mutant islets (Figure 4A). In contrast, 113.8±47.9 pg/mL of IL-1β (n = 4) was detected in the supernatants of palmitate treated macrophages (Figure 4A). To test glucolipotoxicity, islets were treated with 33.3 mM glucose and 1 mM palmitate conjugated to 1% BSA in the presence of 100 nM LPS for 2.5 days. Control islets were treated with LPS and 1% BSA. IL-1β was detectable at extrememly low levels in supernatants from wild-type islets. In NLRP3 mutant islets, this IL-1β concentration was unchanged (Figure S1A). These results indicate that beta-cells lack the ability to produce sufficient IL-1β that could make a noticeable contribution to glucose plus palmitate-mediated cell death.

### Beta-cell specific NLRP3 activating mutation does not cause caspase-1 cleavage in islets

Finally, we examined if intrinsic NLRP3 activation could cause caspase-1 cleavage and, therefore, result in pyroptosis of beta cells. Pro-caspase-1 was detected in LPS treated islets, but addition of glucose and palmitate to the culture medium did not induce its cleavage in either wild-type islets or those expressing mutant NLRP3 (Figure 4B). In contrast, caspase-1 cleavage was observed in macrophages primed with LPS and then treated with glucose and palmitate, or nigericin as positive control (Figure 4B). DNA fragmentation is a feature of pyroptosis and we did not previously see a reduction in DNA fragmentation in caspase-1-deficient islets (Figure 2B–E). To confirm this we used an alternative cell death assay to measure glucolipotoxicity in islets by measuring lactate dehydrogenase (LDH) concentration. Treatment with glucose and palmitate for 2.5 days resulted in increased LDH concentration in the supernatant, but cell death was similar in wild-type as well as mutant NLRP3 islets (Figure S1B). These findings indicate that glucolipotoxicity does not induce pyroptosis of beta cells.

## Discussion

In this study, we have investigated whether exposure of islets to stress could cause inflammasome mediated islet cell death. We did not find a significant role for the NLRP3 inflammasome or IL-1β in islet cell killing in response to high glucose concentrations or chemicals that induce ER or oxidative stress. Previous studies showed that exposure of human islets to 33.3 mM glucose for 5 days resulted in a very low level of IL-1β secretion [9], [12]. Similarly in mouse islets, high glucose concentrations or the ER stress-inducing drug thapsigargin caused a modest amount of IL-1β production (20–40 pg/mL) that was abrogated in islets lacking NLRP3 or TXNIP [13], [19]. These studies all suggested that the IL-1β produced as a result of incubation with glucose or thapsigargin was responsible for islet cell killing. However, the crucial experiment of incubating islets lacking inflammasome components or IL-1 receptors with the toxic stimulus was not done. When we performed this experiment, we found that loss of functional inflammasome factors, NLRP3 or caspase-1, did not inhibit glucose toxicity, ER stress or oxidative stress-mediated death of islet cells. These findings agree with previous data demonstrating that islets lacking IL-1 receptors are not protected from glucose toxicity [6]. Consistent with our results, another study showed that treatment of isolated human islets with high glucose concentration for up to 7 days neither induced IL-1β secretion nor affected IL-1β gene expression [25]. Our data are also in agreement with the concept that mouse islets are not susceptible to killing by IL-1β on its own, but require the addition of IL-1β+IFNγ to induce significant cell death [36]–[38].

Nevertheless, differences in the source (human vs. mouse) of islets, the number of islets, culture conditions, drug concentrations, differences between various beta-cell lines and assays used to measure cell death and inflammasome activity can not be ruled out as possible explanations for the differences between our data and results from some of the previous studies. For example, Maedler and colleagues cultured human islets for 2 days on extracellular matrix coated plates allowing the cells to attach and spread before treating islets with glucose that led to IL-1β secretion [12]. On the other hand we, and others, cultured islets in non-coated petri dishes and treated them with various reagents after 24 hours of isolation [25]. The DNA fragmentation assay we used in our experiments has the advantage that we analyze 10,000 cells per sample, which makes this a highly sensitive method for picking up subtle differences in treatments or genotypes. Although trypsinizing cells does induce some cell death (background DNA fragmentation always below 10%), the proportion of dead cells as a result of trypsinizing was consistent across all treatment groups. Nevertheless, we cannot rule out the possibility that small increases in islet cell death induced by IL-1β could have been masked by cell death induced by trypsinization. Another caveat is the use of LPS in our study to induce signal 1 in islets, where it is possible that other agents, such as Pam2CSK4 may provide a better signal [29]. The story may be further complicated during diabetes *in* *vivo* during which macrophages recuited to the islet could increase IL-1β production [24].

Compared to macrophages, the expression of NLRP3 inflammasome components is very low in islet beta cells [13], and beta cells produced only a modest amount of IL-1β after glucose treatment [13]. Even when we used islets with beta-cell specific expression of a NLRP3 activating mutation, IL-1β production was almost undetectable and glucose toxicity remained similar to wild-type islets. While islet resident macrophages may be a potential source of islet IL-1β, deficiency of either NLRP3 or caspase-1, both components of the NLRP3 inflammasome required for IL-1β processing in macrophages, did not affect *in* *vitro* glucose toxicity mediated killing of whole islets, suggesting the contribution of resident macrophage IL-1β is not significant in this context.

Animal and human studies have shown that high glucose concentration induces oxidative and ER stress in islets [10], [11], [35], [39]–[41]. Increased mitochondrial ROS production in non-islet cells such as THP1 cells or bone marrow derived macrophages (BMDMs) caused inflammasome activation and IL-1β release [20]. Similarly, IL-1β release from glucose treated islets was inhibited by treatment with ROS inhibitors [13]. ER stress induced by reagents such as thapsigarin and tunicamycin also caused IL-1β release from non-islet cells, beta cell lines and primary islets [19], [21]. In addition to IL-1β release, TXNIP was upregulated by these chemicals. These studies concluded that ER and oxidative stress induced inflamasome activation in islets is mediated by TXNIP [13], [19], [21], however, a direct link between TXNIP and inflammasome activation was not formally demonstrated in islets. Treatment of wild-type BMDMs with islet amyloid poplypeptide (IAPP) caused IL-1β production and this was inhibited by NLRP3 deletion but loss of TXNIP had no effect [18]. Our findings that islets lacking functional NLRP3 or caspase-1 are not protected from thapsigargin or rotenone, and previous findings that TXNIP-deficient islets were not protected from thapsigargin or palmitate [22] indicate that TXNIP dependent inflammasome activation may not be important for islet toxicity. TXNIP-deficiency did protect islets from glucose toxicity [22], but this may be due to increased antioxidant activity of thioredoxin in the absence of TXNIP, independent of any effects on the inflammasome.

In conclusion, our results clearly show that activation of the inflammasome does not mediate islet cell death in response to high glucose concentrations, ER or oxidative stress. It could be possible that islets do produce small amounts of IL-1β in response to these stimuli, but by itself this is not enough to cause substantial death *in* *vitro*. Small amounts of IL-1β may increase the expression of chemokines such as CXCL1 in beta cells, thereby facilitating the recruitment of immune cells in the islets [42]. More work will be required to determine if inflammasome activation downstream of glucose toxicity or ER stress has any adverse effects on beta cell function including insulin biosynthesis and glucose stimulated insulin release. In type 2 diabetes, there is an increased concentration of substances such as glucose, saturated and unsaturated fatty acids, IAPP, and circulating cytokines. It is likely that these are also able to induce IL-1β production by macrophages in adipose tissue and other tissues such as islets. However, our data show that while these circulating factors affect islet viability directly, it is less likely that they cause islet cell death through production of IL-1β in a beta cell autonomous manner.

## Supporting Information

Figure S1 **Activation of NLRP3 in beta cells does not alter IL-1β production and cell death. (A)** Four hundred islets/sample were isolated from the mice of indiacted genotypes and cultured in 1 mL of medium containing 100 nM LPS, 33.3 mM glucose and 1 mM palmitate conjugated to 1% BSA for 2.5 days. IL-1β secretion into supernatant was quantified by ELISA. Control islets were incubated in a medium containing 5.5 mM glucose, 1% BSA and 100 nM LPS. n = 1 experiment. **(B)** Cells were treated as in A, then LDH concentration in the supernatant was quantified by LDH assay. Control islets were incubated in a medium containing 5.5 mM glucose, 1% BSA and 100 nM LPS. n = 1 experiment.
(TIF)
