# Supplementary figures and images for: Activation of the NLRP3 Inflammasome Complex is Not Required for Stress-Induced Death of Pancreatic Islets
Source: PLoS One. 2014 Nov 18;9(11):e113128. doi: 10.1371/journal.pone.0113128 (PMC4236141; doi:10.1371/journal.pone.0113128)

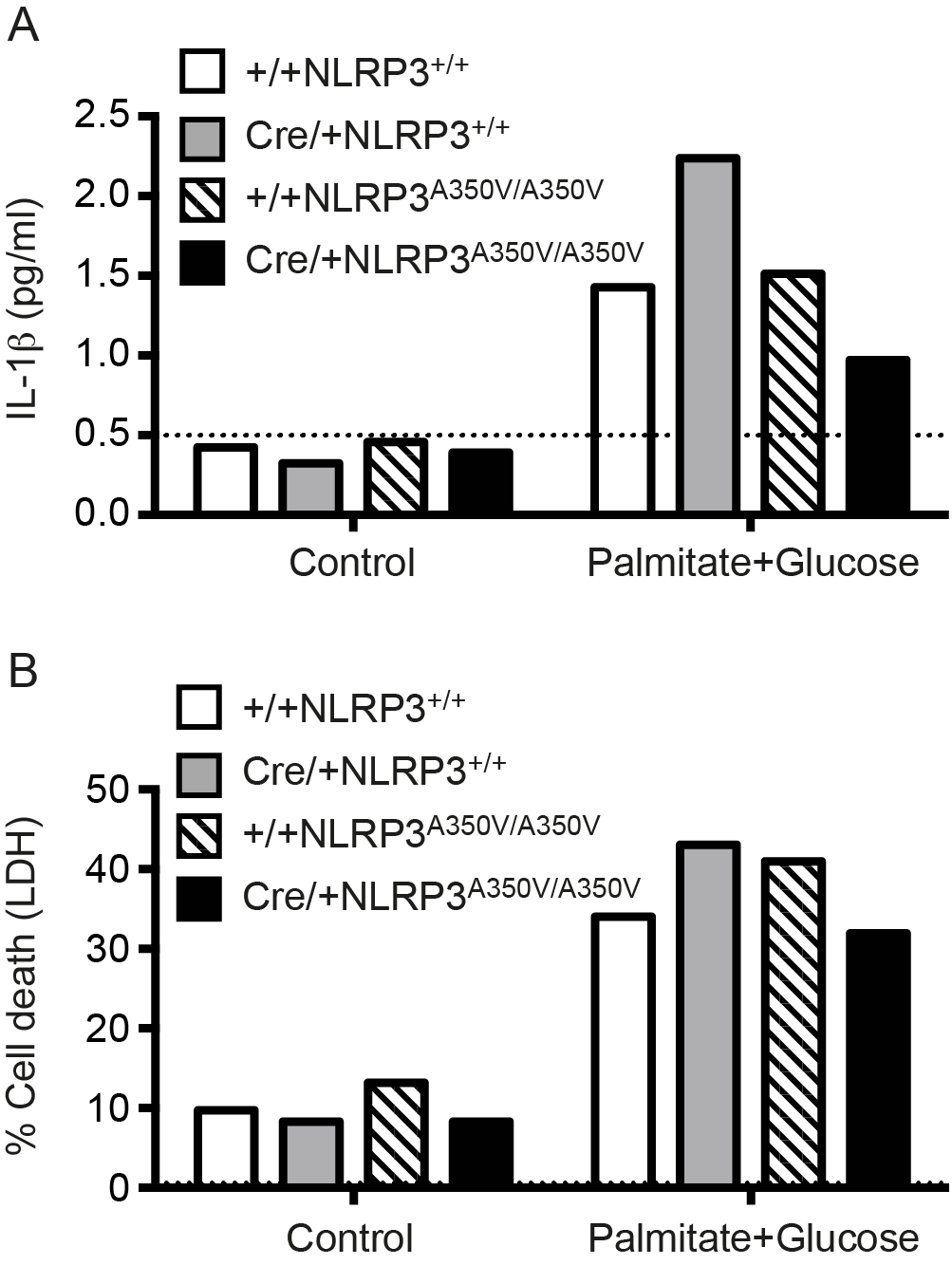

Supplement: Figure S1 — Activation of NLRP3 in beta cells does not alter IL-1β production and cell death. (A) Four hundred islets/sample were isolated from the mice of indiacted genotypes and cultured in 1 mL of medium containing 100 nM LPS, 33.3 mM glucose and 1 mM palmitate conjugated to 1% BSA for 2.5 days. IL-1β secretion into supernatant was quantified by ELISA. Control islets were incubated in a medium containing 5.5 mM glucose, 1% BSA and 100 nM LPS. n = 1 experiment. (B) Cells were treated as in A, then LDH concentration in the supernatant was quantified by LDH assay. Control islets were incubated in a medium containing 5.5 mM glucose, 1% BSA and 100 nM LPS. n = 1 experiment. (TIF) [file pone.0113128.s001.tif]
